# The relationship between college students’ alexithymia and mobile phone addiction: Testing mediation and moderation effects

**DOI:** 10.1186/s12888-018-1891-8

**Published:** 2018-10-11

**Authors:** Songli Mei, Gang Xu, Tingting Gao, Hui Ren, Jingyang Li

**Affiliations:** 1 Department of Social Medicine and Health Management, School of Public Health, Jilin University, NO. 1163 Xinmin Street, Changchun, Jilin Province China; 2 Department of Mental Health, The First Hospital of Jilin University, NO. 71 Xinmin Street, Changchun, Jilin Province China

**Keywords:** Alexithymia, Mental health, Mobile phone addiction, Only child

## Abstract

**Background:**

To explore the relationship between college students’ alexithymia and mobile phone addiction as well as the mediating effects of mental health and the moderating role of being a single child or not.

**Methods:**

A total of 1034 college students from Changchun were assessed with the Toronto Alexithymia Scale (TAS-20), General Health Questionnaire (GHQ) and Mobile Phone Addiction Index (MPAI).

**Results:**

Alexithymia was positively correlated with mental health and mobile phone addiction. Alexithymia had not only a direct impact on mobile phone addiction but also an indirect impact via mental health. For college students who were not only children, higher levels of alexithymia led to an increase in mobile phone addiction, whereas the influence of alexithymia on mobile phone addiction was much weaker among only children.

**Conclusion:**

Mental health has a partial mediating effect on the relationship between alexithymia and mobile phone addiction, and the relationship was significantly moderated by whether students were only children or not.

## Background

The term alexithymia originates from Greek and literally means “lacking words for emotions” [1]. Alexithymia is a multifaceted construct associated with difficulties in identifying, analyzing and verbalizing feelings, constricted imagination, and a concrete, externally oriented way of thinking [2]. Individuals with alexithymia have limited ability to understand their own feelings and others’ emotions and cannot regulate emotions properly in interpersonal contexts [3]. Research on the absolute and relative stability of alexithymia has shown that alexithymia is a personality trait instead of a state-dependent phenomenon that is secondary to other clinical problems [4]. The most widely used self-reported measure of alexithymia is the 20-item Toronto Alexithymia Scale (TAS-20) [5]. There are three factors for this scale: (1) difficulty in identifying feelings (DIF), (2) difficulty in describing feelings (DDF), and (3) externally oriented thinking (EOT). The prevalence of alexithymia has been shown to range from 13 to 19% [6]. The significant percentage of 24.1% of young people is observed to have high levels of alexithymia [7].

Alexithymia is very common in individuals with psychiatric disorders [8] and is a sign of negative emotion in psychiatric populations [9]. Alexithymia may restrict the control of emotional states and may lead to negative affect, including depression and anxiety [10]. Individuals who suffer from intolerable psychological disease sometimes cannot express themselves by suitable words [11]. It is generally accepted that alexithymia has profound effects on mental health. Compared with non-individuals with alexithymia, individuals with alexithymia were prone to report more mental health-related problems [12]. There was an inverse and strong relationship between alexithymia and mental health; that is, mental health may be strengthened by interventions targeting alexithymia [13].

The mobile phone has gained a strong position in modern life and human society and is regarded as an indicator of communication technology [14]. Despite its convenience for many people, the problems derived from overuse of the mobile phone are a subject of much concern. Mobile phone addiction is characterized by uncontrolled mobile phone use that leads to adverse consequences on an individual’s physical and mental health and social functioning [15]. Individuals with symptoms of mobile phone addiction tend to bring their phone with them wherever they are and think about their phone even if they cannot use it, which ultimately influences daily tasks [16]. Of all applications, instant messaging receives the highest use among mobile phone users in China (92.1%), with 11.0% higher use than the second most common use, the search engine [17]. This shows that individuals use the mobile Internet for social needs more than for other functions. Some users believe that mobile phone instant messaging not only can help them build deeper friendships but also is more comfortable than face-to-face interactions [18]. However, people who overuse mobile phones are more likely to have difficulties expressing their emotions than the general population is [19]. Individuals with mobile phone dependence may have deficient facial expression recognition and take more time to identify types of emotion. Alexithymia is significantly correlated with dysregulation of emotions and affects, which makes it difficult to guide one’s own behavior [20]. Mobile phone addiction among university students could result from pre-existing factors [21]. Alexithymia is considered a high-risk factor for mobile phone addiction [22].

There is a significant negative correlation between mental health and the level of addiction to mobile phone use [23]. Mobile phones can be used to avoid negative emotions, which may magnify such affects because of negative emotional responses and unresolved fundamental problems [24]. Students who have poor mental health and are psychologically unbalanced are more susceptible to engaging in addictive mobile phone behaviors because they attempt to decrease their intense negative emotions by communicating with others [25].

The literature review showed that alexithymia, mental health and mobile phone addiction are correlated with each other. A previous study has shown that alexithymia could mediate the association between self-awareness and anxiety as well as depression [26]. Alexithymia is closely and positively correlated with chronic pain, and negative affects, including depression and anxiety symptoms, mediate this relationship [27]. Mental health might be a mediator in the association between alexithymia and mobile phone addiction.

China has relaxed its more than three-decade-old family planning policy. The implementation of the universal two-child policy is intended to actively address the country’s aging trend. Only children represent a certain proportion in China. Whether one is an only child or not has an influence on alexithymia, and the alexithymia scores of children with a sibling were higher than those of children without [28]. A Chinese study also found that the level of mobile phone dependence among college students who were only children was significantly higher than that among those who were not only children [29]. However, these studies investigated differences in alexithymia and mobile phone addiction only in only children.

The aim of the present study was to determine the potential mechanisms underlying the relationship between alexithymia and mobile phone addiction. Specifically, we will examine how alexithymia influences mobile phone addiction through mental health and differences in the association between alexithymia and mobile phone addiction among only children and children with a sibling. Based on the literature review, we propose the following two hypotheses:

Hypothesis 1: Mental health will mediate the association between alexithymia and mobile phone addiction.

Hypothesis 2: Being an only child or not will moderate the association between alexithymia and mobile phone addiction.

## Method

### Participants

A cross-sectional survey was conducted from April to May 2015. A convenience cluster sampling method was employed to produce a sample of college students. All participants were recruited from Jilin University, a comprehensive university in Northeast China. Everybody involved in this study will be rewarded with credits of some certain courses and told to participant voluntarily and could withdraw at any time. The participants answered a traditional paper-and-pencil questionnaire with the guidance of well-trained researchers during school classes. It took the respondents approximately 30 min to complete the anonymous questionnaire. A small gift was given to make up for the time spent on the survey.

The prevalence of mobile phone addiction among Chinese undergraduates was 21.3%, so π = 21.3%. A relative error of 15% was allowed in the present study. The absolute error can be calculated by δ = 0.15π = 0.15 × 21.3%. We adopt 95% confidence intervals; thus, *μ*_*a*_ = 1.96. According to the following equation for the sample size, we calculated the minimum sample size: n = [1.96^2^ × 21.3% × (1–21.3%)]/(0.15 × 21.3%)^2^ ≈ 631. Considering the invalid cases, the desired sample size should increase by 10%: 631 × (1 + 10%) ≈ 695.

$$ n=\left(\frac{u_a^2\kern0.1em \pi \left(1-\pi \right)}{\delta^2}\right) $$

A total of 1200 college students participated in this survey. After the subjects with missing data were excluded, the sample included 1034 subjects.

### Measures

#### Toronto alexithymia scale (TAS-20)

The Toronto Alexithymia Scale-20 (TAS-20) is a self-report scale for the assessment of alexithymia [5]. The instrument consists of 20 items rated using a five-point Likert scale. The total score ranged from 20 to 100, with higher scores indicating a higher level of alexithymia traits. Research suggests that TAS-20 is appropriate for use with the Chinese population [30]. According to a categorical approach [31], a total of 57 points or more indicates a high level of alexithymia, while the range of values from 40 to 57 indicates a moderate level of alexithymia, whereas and 40 points or below indicates a low level of alexithymia. The Cronbach’s alpha in this study was 0.81.

#### General health questionnaire (GHQ-12)

Mental health was measured with the General Health Questionnaire (GHQ-12) [32]. The GHQ-12 includes 12 items describing mood states over the previous 4 weeks. The original GHQ rating method (0–0–1-1) was used in the questionnaire. The total score ranged from 0 to 12 points, with higher scores indicating poor psychological well-being. Research suggests that the GHQ-12 is appropriate for use with the Chinese population [33].The Cronbach’s alpha in this study was 0.76.

#### Mobile phone addiction index (MPAI)

Mobile phone addiction was measured by the Chinese version of the Mobile Phone Addiction Index (MPAI) [34], which was developed by Leung [35]. This is a self-report questionnaire with 17 items, which rated on a 5-point Likert scale. It contains four subscales: inability to control craving, feeling anxious and lost, withdrawal or escape and productivity loss. Higher scores indicated higher levels of mobile phone addiction. The Cronbach’s alpha in this study was 0.87.

### Data analysis

The descriptive analysis was used to determine the demographic characteristics of the participants. Pearson correlation analyses of the study variables were conducted. We adopt independent-samples T-test to examine differences in mobile phone addiction and mental health between individuals with high and low alexithymia. The structural equation model (SEM) was used to study the effects of alexithymia on mobile phone addiction through mental health. The bootstrapping method was used to verify mediation effects. In this study, we bootstrapped 5000 samples from the data, and 95% bootstrap confidence intervals (CI) were calculated. A hierarchical multiple linear regression was conducted to verify whether being an only child moderated the relationship between alexithymia and mobile phone addiction. Statistical analysis was conducted using the SPSS 18.0 version program and Amos 17.0 software.

## Results

### Sample characteristics

The mean age of the participants was 19.97 years (SD = 1.22). The sample consisted of 1034 college students, of whom 52.7% (*n* = 545) were women. Five hundred forty-three (52.5%) participants were only children. Over half (57.4%, *n* = 594) were from urban areas. A total of 534 individuals (51.6%) had moderate income, and 286 individuals (27.7%) had low family income, while the remainder had high income (20.7%, *n* = 214). Table 1 shows the detail of demographic characteristics of participants.

**Table 1:** Demographic characteristics of the college students

Variables	n	%
Gender		
Male	489	47.3
Female	545	52.7
Single child		
Yes	543	52.5
No	491	47.5
Area of family residence		
Urban	594	57.4
Rural	440	42.6
Grade		
One	348	33.7
Two	563	54.4
Three	123	11.9
Family income status		
Low income	286	27.7
Moderate income	534	51.6
High income	214	20.7

### Bivariate statistics

Means, standard deviations and correlations between all the study variables are presented in Table 2. All the dimensions of alexithymia, mobile phone addiction and poor mental health were positively correlated with each other, while externally oriented thinking was not correlated with withdrawal or escape.

**Table 2:** Means, standard deviations and correlations for all variables (*n* = 1034)

Variables	*M* ± *SD*	1	2	3	4	5	6	7	8
1. Difficulty in Identifying Feelings	17.28 ± 5.14	1							
2.Difficulty in Describing Feelings	13.23 ± 3.22	0.68^**^	1						
3. Externally Oriented Thinking	20.05 ± 3.94	0.28^**^	0.31^**^	1					
4. Inability to Control Craving	14.89 ± 4.85	0.32^**^	0.27^**^	0.19^**^	1				
5. Feeling Anxious and Lost	12.11 ± 4.87	0.19^**^	0.16^**^	0.07^*^	0.47^**^	1			
6. Withdrawal or Escape	7.57 ± 3.11	0.20^**^	0.15^**^	0.05	0.37^**^	0.43^**^	1		
7. Productivity Loss	5.24 ± 2.20	0.29^**^	0.24^**^	0.13^**^	0.59^**^	0.37^**^	0.39^**^	1	
8. Mental Health	3.20 ± 2.60	0.44^**^	0.37^**^	0.23^**^	0.30^**^	0.22^**^	0.15^**^	0.30^**^	1

Note:^**^P<0.01, ^*^P<0.05

**Table 3:** Comparison of mobile phone addiction and mental health by alexithymia

Variables	High alexithymia	Low alexithymia	*t*
Inability to Control Craving	17.36 ± 5.30	12.74 ± 4.20	−9.44^***^
Feeling Anxious and Lost	13.65 ± 5.12	11.55 ± 5.17	−4.15^***^
Withdrawal or Escape	8.25 ± 3.30	6.78 ± 3.36	−4.46^***^
Productivity Loss	6.12 ± 2.20	4.31 ± 2.30	−8.16^***^
Mobile Phone Addiction	45.38 ± 12.16	35.39 ± 11.53	−8.46^***^
GHQ	5.08 ± 2.97	1.96 ± 1.80	−11.95^***^

Note:^***^P<0.001

#### Comparison for mobile phone addiction and mental health by alexithymia

The results showed that there were significant differences in mobile phone addiction and mental health between individuals with high and low alexithymia (*P* < 0.001).The mobile phone addiction and mental health scores of individuals with high alexithymia were significantly higher than those of individuals with lower alexithymia. The higher level of alexithymia an individual had, the greater the possibility of mental health problems and mobile phone addiction (Table 3).

#### Mediation analysis of alexithymia on mobile phone addiction

To determine the relationship between alexithymia, mental health and mobile phone addiction, we first created a direct model of alexithymia on mobile phone addiction according to the stepwise regression method. Second, we created mental health as the mediator variable and built the mediation model of mental health on the relationship between alexithymia and mobile phone addiction. The results of the hierarchical regression on the mediation model test were as follows. After controlling for gender and being an only child, the path coefficient of alexithymia on mobile phone addiction was 0.316 (*t* = 10.694, *P* < 0.001) in the direct path model, and the explanation rate was 10.9% (R^2^ = 0.109). After mental health was included as the mediator variable, the direct path coefficient of alexithymia on mobile phone addiction was reduced to 0.218 (*t* = 6.749, *P* < 0.001). The explanation rate of the mediation model for mobile phone addiction variance increased by 14.7% (R^2^ = 0.147). This result indicated that the mediation model was superior to the direct path model. The inclusion of mental health (mediator variable) can explain the greater variance in mobile phone addiction, which can better explain the relationship between alexithymia and mobile phone addiction and play a partial mediating role in the relationship.

**Fig. 1:**
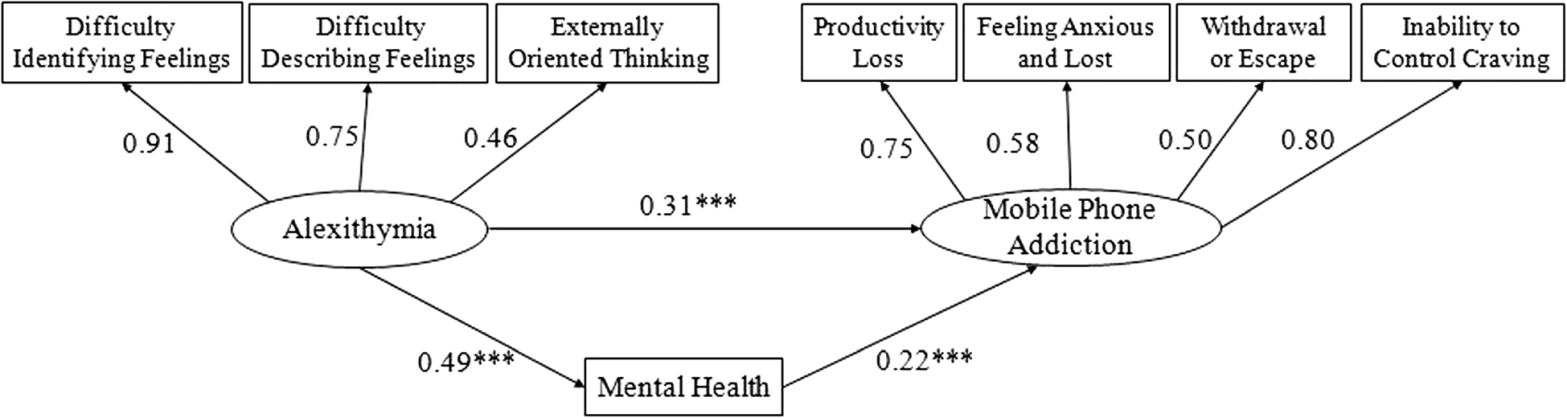
Model for alexithymia, mental health and mobile phone addiction

Alexithymia was used as the predictor variable, mobile phone addiction as the outcome variable, and mental health as the mediating variable to build the SEM by Amos 17.0 software. The fit indices of the model yielded satisfactory results (χ^2^/*df* = 1.384, NFI = 0.992, TLI = 0.995, CFI = 0.998, RESEA = 0.019). The result of the path coefficients is presented in Fig. 1.

To gain an improved understanding of the mediation effects, we performed the mediation tests by applying the bootstrapping procedure. We drew 5000 bootstrapping samples and computed 95% confidence intervals (95%CI). We made alexithymia the predictor variable, mobile phone addiction the outcome variable and mental health the mediating variable. Then, we included the study variables in the PROCESS macro for SPSS. The results demonstrated that the total effect of the model was 0.115. The confidence interval was excluding zero (LLCI = 0.075, ULCI = 0.157), which showed that a reasonable mediation model was established. The mediation effects of mental health could explain 13.7% (R^2^ = 0.137) of the total variance. Thus, alexithymia exerted a significant indirect effect on mobile phone addiction via mental health.

### Moderation analysis of being an only child

**Fig. 2:**
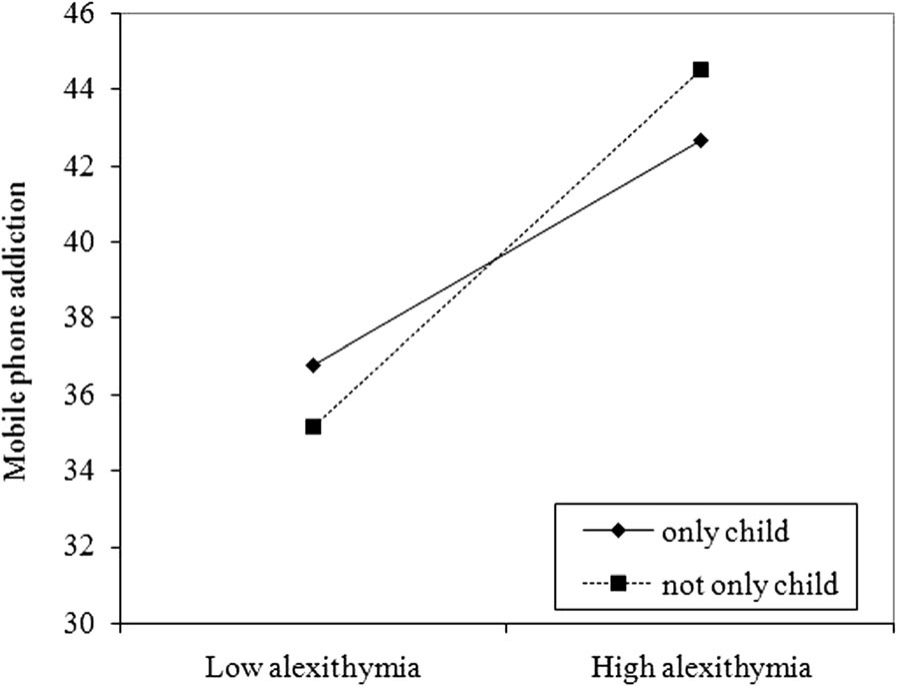
The moderating effect of being an only child or not on the relationship between alexithymia and mobile phone addiction

A hierarchical multiple linear regression was conducted to verify whether being an only child moderated the relationship between alexithymia and mobile phone addiction. Because only children belonged to two categorical variables and alexithymia was a continuous variable, we needed to apply grouping regression analysis to test the moderation analysis, according to the suggestion of Wen [36]. Above all, alexithymia as the continuous predictor was centered. A dummy variable represented whether children were only children. The interaction term of alexithymia and being an only child or not was obtained at the same time. Then, mobile phone addiction was used as the outcome variable through hierarchical multiple linear regression. First, alexithymia and being an only child or not were entered in Step 1. Second, the interaction term of alexithymia and being an only child or not was entered in Step 2. The results showed that R_1_^2^ was significantly higher than R_2_^2^(△R^2^ = 0.006, △F = 6.818,*P* < 0.01), which explains the moderation effect of whether being an only child or not was significant. To investigate the improved mechanism of the mediation effects, we performed a regression analysis of alexithymia and mobile phone addiction for only children and for not only children. The results showed that alexithymia was positively associated with mobile phone addiction among college students who were only children (*β* = 0.259, R^2^ = 0.067, *F* = 39.295, *P* < 0.001) and those who were not children (*β* = 0.372, R^2^ = 0.139, *F* = 79.105, *P* < 0.001). Simple slope tests showed that for college students who were not only children, higher levels of alexithymia led to an increase in mobile phone addiction (*β* = 0.482, *P* < 0.001). However, for college students who were only children, the effect of alexithymia on mobile phone addiction was much weaker (*β* = 0.304, *P* < 0.001) (Fig. 2).

## Discussion

Our finding of a positive relationship between alexithymia, poor mental health and mobile phone addiction is consistent with the existing literature [19, 37, 38]. The present study also extended previous work by showing that scores of mobile phone addiction and GHQ in high alexithymia was significantly higher than in low alexithymia. Compared to individuals without alexithymia, individuals with alexithymia were subject to a higher potential risk for Internet addiction [22]. The most obvious reason for this association is that individuals with alexithymia attempt to regulate their emotions by addictive behavior [39]. However, a previous study has argued that individuals with social relationship problems that might be caused by alexithymia showed less frequent mobile phone use [40]. In general, the mental health of individuals with alexithymia was poor when compared to that of individuals without alexithymia [41]. A meta-analysis indicated that alexithymia served as a critical path to different indicators of mental health [42]. Alexithymia has direct effects on the inability to experience emotions, leading to poor mental health. The hierarchy of needs theory proposed by Maslow holds that an individual’s mental health is closely related to the satisfaction of a need. If an individual’s needs are not met, negative mentality may be more easily generated and cause psychological problems [43]. Based on the associated expectations of anonymity, convenience and avoidance, individuals use the mobile phone to obtain psychological and physiological satisfaction. According to the theory of use and gratification, long-term mobile phone use will form mobile phone addiction.

According to the results of the mediation test, mental health mediated the relationship between alexithymia and mobile phone addiction, which supports Hypothesis 1. To our knowledge, this is the first study to investigate the mechanism among alexithymia, mental health, and mobile phone addiction in a sample of college students. Alexithymia had not only a direct impact on mobile phone addiction but also an indirect impact via mental health. College students are in a period of the rapid development of social consciousness. They pay more attention to their own values and inner world, and hope to receive attention from others and integrate into their peer groups. When these needs are not met, mobile phones, the Internet and other new media can help students achieve the impression of communication and share their feelings. Mobile phones are a convenient and popular tool for contacting others [40]. An increasing body of studies has provided evidence of the association between alexithymia and Internet addiction [44]. Individuals with alexithymia may use the Internet to express their feelings as a compensatory, nonverbal strategy [1]. The opportunity to gain better control over the communication process can help individuals with alexithymia manage their moods, regulate their emotions during social interactions and find a more effective means of communication that suits them [45]. Individuals with alexithymia have difficulty gaining enough resources to face stressful life events, which causes highly negative evaluations of coping style. Individuals with alexithymia may seek to relieve the poor mental health due to alexithymia by turning to mobile phones and therefore may be more prone to excessive use of mobile phones.

In addition, our results showed that the association between alexithymia and mobile phone addiction was moderated by being an only child or not, which supports Hypothesis 2. More specifically, for college students who were not only children, higher levels of alexithymia led to an increase in mobile phone addiction, whereas the effect of alexithymia on mobile phone addiction was much weaker among only children. This may be because children with siblings face competition for all types of resources from their brothers and sisters. To attract their parents’ attention, it is easy to establish an inappropriate defense mechanism and then avoid inner emotional cognition. The basic function of the mobile phone makes the phone a better means to seek peer recognition and improve one’s sense of belonging. When only children grow up in a relatively relaxed and wealthy economic condition, they are more willing to express and examine their inner feelings, and the risk that they will depend on mobile phones to meet their emotional demands is low.

### Limitations

Because the present study was based on a self-reported questionnaire, there might be many associated confounding factors. In addition, a cross-sectional survey cannot be used to draw a definitive conclusion. Thus, further study requires a longitudinal design. In addition, only one university was involved in this study, which could affect the generalizability of the findings. Lastly, in addition to the influence of mental health and being an only child or not, there must be other variables affecting the relationship between alexithymia and mobile phone addiction that need to be further discussed.

## Conclusion

The present study verified the mediating effect of mental health and the moderating effect of being an only child or not between alexithymia and mobile phone addiction. Mental health and individual characteristics should be considered when relevant departments design strategies for the prevention of mobile phone addiction.

## Abbreviations

CI: Confidence intervals
SEM: Structural equation model
